# Optimizing legume green manure applications for enhanced forage sorghum-sudangrass performance and soil property improvements

**DOI:** 10.7717/peerj.20137

**Published:** 2025-10-03

**Authors:** Emre Kara

**Affiliations:** Faculty of Agriculture, Adnan Menderes University, Aydın, Turkey

**Keywords:** Green manure, Incorporation time, Forage quality, SPAD, NDVI, LAI, Sorghum-sudangrass

## Abstract

**Background:**

Legume-based green manuring is an environmentally sustainable and economically viable approach that enhances soil fertility by improving organic matter content, facilitating biological nitrogen fixation, and stimulating microbial activity. These benefits collectively reduce reliance on mineral fertilizers, which are associated with environmental degradation. While the soil-enhancing properties of legumes are well-established, limited information is available regarding how the timing of green manure incorporation affects the growth performance and forage quality of subsequent crops. This study aimed to assess the effects of incorporating different legume species at various phenological stages on the growth, yield, and forage quality of sorghum × sudangrass under Mediterranean climatic conditions.

**Methods:**

A field experiment was conducted from 2022 to 2024 at Aydın Adnan Menderes University, Türkiye, using a randomized complete block design with three replicates. Eleven treatments were implemented, consisting of three legume species (common vetch, narbon vetch, and forage pea) incorporated at three distinct phenological stages (pre-flowering, 10% flowering, and full flowering), along with unfertilized and fertilized controls. Following incorporation, sorghum × sudangrass was sown without additional fertilization, except in the fertilized control. Key response variables included fresh forage yield (FFY), hay yield (HY), plant height, leaf number, leaf area index (LAI), chlorophyll content (SPAD), and crude protein yield (CPY). Soil organic matter and total nitrogen levels were also measured before and after treatments.

**Results:**

Green manure application significantly enhanced the growth, biomass accumulation, and forage quality of sorghum × sudangrass. The incorporation of common vetch and narbon vetch at the 10% flowering stage led to up to a 50% increase in fresh forage yield compared to the unfertilized control. Forage pea maintained consistent yield performance across all incorporation stages. Improvements in soil organic matter and nitrogen content were particularly notable in legume-amended plots. Furthermore, the highest SPAD and LAI values were recorded at early flowering stages, suggesting enhanced photosynthetic efficiency. These findings underscore the effectiveness of legume green manuring, particularly with optimal incorporation timing, as a sustainable strategy to improve soil health, forage productivity, and resource-use efficiency in Mediterranean agroecosystems.

## Introduction

Modern agriculture is increasingly confronted with the dual imperative of sustaining crop productivity and ensuring long-term environmental sustainability (Crews, Carton & Olsson, 2018; Shah & Wu, 2019). The intensive and prolonged use of mineral fertilizers, while effective for short-term yield enhancement, has contributed to a range of environmental problems, including greenhouse gas emissions, nitrate leaching, soil degradation, and biodiversity loss (Verma, Pramanik & Bhaduri, 2019; Tripathi et al., 2020). In light of these concerns, attention has shifted toward alternative soil fertility management strategies that maintain productivity while preserving ecological integrity. Among the most widely studied options, green manuring with leguminous crops has gained renewed importance as a cost-effective and ecologically sound approach to sustaining soil fertility, supporting nutrient cycling, and enhancing agroecosystem resilience in the face of climatic and resource limitations (Meena et al., 2018; Kumar et al., 2020). These systems offer multiple agronomic benefits, notably biological nitrogen fixation, increased soil organic matter, and enhanced microbial activity, while also contributing to weed suppression and improved nutrient use efficiency (Ma et al., 2021; Gao et al., 2016; Pu et al., 2023). As a result, leguminous green manures are gaining traction as reliable tools for sustainable and adaptive cropping systems.

Yield-enhancing effects of green manure have been widely documented in crops such as maize, rice, and sunflower. Enhanced nitrogen supply from decomposing legume residues leads to improved biomass accumulation and nutrient uptake (Özyazıcı & Manga, 2000; Bai et al., 2017; Santos et al., 2018; Ye et al., 2023). At the same time, the incorporation of legumes provides significant environmental benefits, including reduced soil erosion, enhanced soil water retention capacity, and a decrease in nitrate leaching into groundwater. These benefits are especially vital in areas experiencing climatic instability and soil degradation, where protecting soil health and reducing nutrient losses are essential for sustainable agricultural systems (Li et al., 2021; Zhao, 2023).

Despite these well-documented advantages, a specific knowledge gap remains regarding the optimal timing of legume green manure incorporation, particularly in forage-based cropping systems and Mediterranean climates. While prior studies have examined the effects of species type or biomass quantity, few have explored how incorporation timing affects system-level processes. The phenological stage at which legume green manure is incorporated is central to regulating nutrient cycling, microbial activity, and soil physical properties, all of which ultimately influence crop performance. Residue maturity affects decomposition dynamics through changes in C:N ratio and lignin content, thereby altering nitrogen availability, water use efficiency, root development, and soil moisture retention. As such, optimizing incorporation timing is essential to synchronize nutrient release with crop demand and enhance overall system efficiency (Naaiik, Mishra & Madhavi, 2019; Tanwar, 2023). This issue is especially relevant in Mediterranean regions, where water scarcity, erratic rainfall, and shortened growing seasons present increasing challenges for forage production. These constraints necessitate more precise organic matter management, particularly in aligning the decomposition of green manure with the nutrient demands of the main crop. If incorporation occurs too early, nutrients may be lost before uptake. Conversely, delayed incorporation can result in residues with higher lignin content and slower decomposition, reducing their immediate benefit. Understanding how incorporation timing affects soil fertility and crop response is thus essential for designing adaptive, resource-efficient forage production systems (Rule, Turley & Vaidyanathan, 2003).

In Mediterranean agriculture, forage legumes such as common vetch (*Vicia sativa*), narbon vetch (*Vicia narbonensis*), and forage pea (*Pisum sativum* subsp. *arvense* (L.) Asch. & Graebn.) are widely used as cover crops and seasonal forage. These species improve soil nitrogen and carbon pools, enhance microbial processes, and support efforts to reduce erosion and stabilize soils (Neely, 2024; Neely et al., 2024). When incorporated at the right stage, they provide substantial nitrogen inputs to subsequent crops, such as sorghum and its hybrids, thereby supporting high biomass production and forage quality with reduced reliance on synthetic fertilizers (Barros et al., 2020; Kara, Sürmen & Erdoğan, 2019; Al-Chammaa, Al-Ain & Kurdali, 2019).

In addition to nutrient dynamics, water-use efficiency (WUE) is increasingly recognized as a key determinant of forage performance under rising climatic stress in arid and semi-arid zones. This is consistent with previous studies reporting that residue maturity affects microbial and root-related soil processes (Naaiik, Mishra & Madhavi, 2019; Tanwar, 2023). Legume residues rich in nitrogen decompose rapidly, enhancing nutrient mineralization and improving soil porosity, infiltration, and water-holding capacity. Deep-rooted legumes further access subsoil water, supporting forage crops during dry spells (Barros et al., 2020; Al-Ain, Al-Chammaa & Kurdali, 2017; Carvalho et al., 2015).

One crop with strong potential to benefit from improved green manure strategies is sorghum × sudangrass (*Sorghum bicolor* × *Sorghum sudanense*), a drought-tolerant forage hybrid that has been widely adopted in hot and dry regions such as Türkiye. Due to its heat tolerance and efficient resource use, sorghum × sudangrass can maintain photosynthesis and biomass production under severe stress conditions, making it an ideal component of climate-resilient forage systems (Sürmen & Kara, 2022; Alatürk, 2024; Schittenhelm, 2010; Yartsev et al., 2024; Khalifa & Eltahir, 2023). Additionally, its root structure improves soil aggregation and contributes to carbon sequestration (Hasan et al., 2015; Indu et al., 2022; Sustek-Sánchez et al., 2023).

However, there is a lack of systematic research evaluating how different green manure species and incorporation stages affect sorghum × sudangrass yield and forage quality under Mediterranean conditions. Existing studies often neglect phenological variation or are limited to grain crops rather than high-biomass forage systems. Given the increasing variability in rainfall and growing conditions, optimizing green manure timing is crucial for synchronizing nutrient release, improving water use, and strengthening system resilience.

This study aims to address this gap by evaluating the effects of three legume species (common vetch, narbon vetch, and forage pea) incorporated at three phenological stages. Findings from this research will provide practical insights for farmers and agronomists seeking to optimize forage production with minimal external inputs under increasing climate pressures. Moreover, it contributes to the development of climate-smart and region-specific green manure strategies that align soil regeneration with long-term yield sustainability in Mediterranean forage systems.

## Materials and Methods

### Site description and climate

The experiment was established at the Aydın Adnan Menderes University Research and Application Farm, located in Koçarlı district, Aydın province, Türkiye (37°44′N, 27°45′E, at an elevation of 25–65 m above sea level). The field is situated in the Büyük Menderes Basin, where the Mediterranean climate prevails. Before the establishment of the experiment, the land had been utilized for rotational cultivation of various leguminous and grass forage crops until 2022. The study was carried out from October 2022 to September 2024 to assess the impact of green manure applications on forage crop performance and associated soil properties. To evaluate the effects of different green manure treatments, three forage legume species, common vetch (*Vicia sativa* L.), narbon vetch (*Vicia narbonensis* L.), and forage pea (*Pisum sativum* subsp. *arvense (*L.) Asch. & Graebn.) were tested alongside fertilized and unfertilized control plots. The soils at the experimental site are young alluvial deposits with moderate lime content, classified as Inceptisols under the Soil Taxonomy. These soils fall under the Xerept suborder, which exhibits relatively weak horizon development, and their organic matter content and total nitrogen levels range from low to moderate. Their susceptibility to waterlogging, particularly in winter, and the presence of stoniness are significant factors that limit their agricultural use. According to the FAO/WRB classification, these soils are identified as Hypocalcic Calcisol, where high lime content may limit the availability of plant nutrients, particularly phosphorus (Mert Akça & Atatanır, 2020). Soil samples taken before green manure incorporation revealed the following properties: a pH of 8.09, available phosphorus of 5.11 ppm, exchangeable potassium of 197 ppm, exchangeable calcium (Ca) of 4,301 ppm, iron (Fe) of 14.94 ppm, zinc (Zn) of 0.35 ppm, and a sandy loam texture.

**Figure 1:**
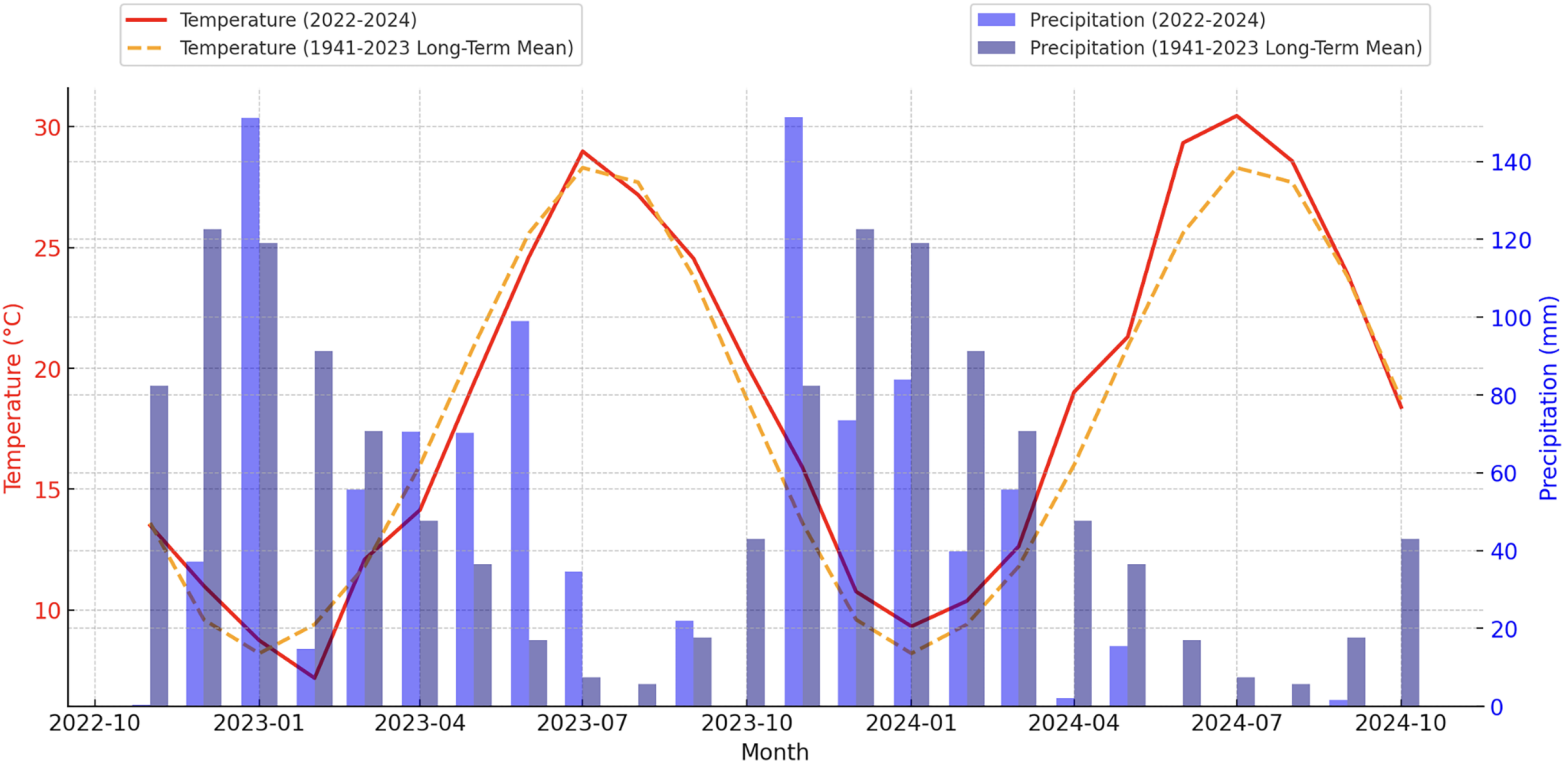
Monthly temperature and precipitation trends (2022–2024) in the experimental area compared to long-term averages (1941–2023).

Temperature data from 2022 to 2024 indicated notable deviations from long-term averages. While temperatures in January and February remained below long-term averages (8.2 °C and 9.4 °C, respectively), March recorded 12.09 °C, exceeding the long-term mean of 11.8 °C. In terms of precipitation, January recorded 151.2 mm, surpassing the long-term average of 119.1 mm, whereas February precipitation dropped sharply to 14.8 mm, significantly below the average of 91.3 mm. The combination of low winter temperatures and reduced precipitation suggests an increased risk of drought, while relatively high spring temperatures reflect the regional impacts of seasonal variability (Fig. 1).

**Figure 2:**
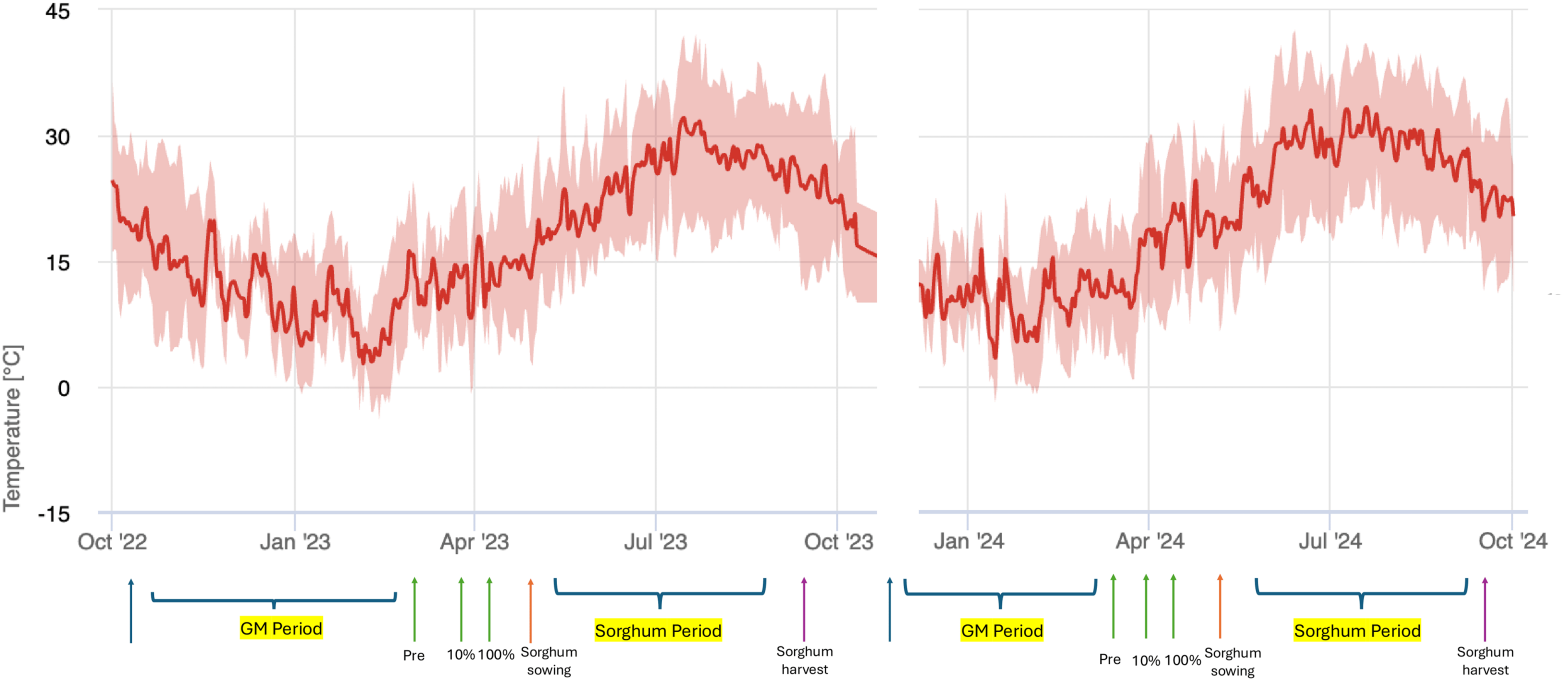
Daily temperature variability and key agricultural periods (2022–2024) in the experimental area. (FieldClimate by Pessl Instruments, iMetos 2): Maximum, Minimum, and Mean Trends of Green Manure and Sorghum Growth Stages.

Notably, maximum summer temperatures frequently ranged between 35°C and 40°C, surpassing long-term averages. This trend reflects a rising frequency of extreme heat events, thereby increasing the region’s susceptibility to drought stress (Fig. 2).

### Experimental design and agronomic management

Treatments for the field experiment were arranged in a randomized complete block design with three replicates. The study included five winter treatments, consisting of various legume-based green manure strategies and controls:

(1) Winter fallow – unfertilized sorghum × sudangrass (cv. Tonka),
(2) Winter fallow – fertilized sorghum × sudangrass,
(3) Common vetch (cv. Alper) + sorghum × sudangrass,
(4) Narbon vetch (cv. Bozdag) + sorghum × sudangrass,
(5) Forage pea (cv. Taskent) + sorghum × sudangrass.

Each green manure treatment was further combined with three phenological incorporation stages: pre-flowering, 10% flowering, and 100% flowering. The incorporation stage was selected based on previous findings that demonstrate its influence on nutrient release and subsequent crop uptake (Morelli et al., 2022).

No fertilization was applied prior to the sowing of green manure crops. *Rhizobium* inoculation was not performed because the experimental field had been previously cultivated for 13 consecutive years with various leguminous forage species, during which effective nodulation had been consistently observed. As a result, it was assumed that sufficient populations of native *Rhizobium* were already present in the soil, making additional inoculation unnecessary.

The seeding rates were determined as 100 kg ha^−1^ for common vetch and 150 kg ha^−1^ for narbon vetch and forage pea. No pesticides were applied during the green manure growth period. The study was conducted with 11 different treatments, each replicated three times. Each plot measured 28 m^2^ (8 × 3.5 m), and this plot configuration was consistently used for both the green manure and subsequent sorghum × sudangrass phases. The row spacing was set at 25 cm for green manure and 50 cm for sorghum × sudangrass. The aboveground biomass of the green manure crops was first mowed using a mechanical mower. Subsequently, the residues were incorporated into the soil using a plot-type rototiller (Yurduser Agr., İzmir, Türkiye) to a depth of approximately 15–25 cm. To ensure thorough and uniform incorporation, the tillage process was performed twice. Twenty days after incorporation at the 100% flowering stage, sorghum × sudangrass was sown using a seed drill (Maschio Gaspardo, Italy). Following the green manure and control treatments, no fertilization was applied to sorghum × sudangrass plots. However, in the fertilized sorghum × sudangrass treatment, a two-stage fertilization strategy was implemented. Basal fertilization consisted of 50 kg ha^−1^ of nitrogen and 50 kg ha^−1^ of phosphorus, applied using a compound fertilizer (20-20-0, N-P_2_O_5_-K_2_O). Topdressing fertilization included 50 kg ha^−1^ of nitrogen, applied as ammonium sulfate ((NH_4_)_2_SO_4_). Irrigation was applied uniformly across all plots using a drip irrigation system. Water applications were scheduled based on calculated soil field capacity, ensuring that moisture levels remained within 70–80% of field capacity to avoid both drought stress and excess leaching. Soil moisture was monitored regularly to determine irrigation timing and amounts, and adjustments were made to maintain optimal conditions for both green manure and sorghum × sudangrass growth. Sorghum × sudangrass was harvested uniformly by cutting at the milk-dough stage in both measurement years to an average stubble height of 10 cm. To minimize edge effects, samples for dry matter content and chemical analyses were taken from the three central rows after mowing, while 50 cm was left unharvested at both the front and rear ends of each plot, and the outermost rows on both sides were excluded from sampling.

### Measurements

Green manure biomass was measured using 1 × 1 m quadrats immediately before incorporation. Total fresh forage yield (t ha^−1^) was recorded at the harvest of sorghum × sudangrass in each plot. A subsample was then oven-dried at 78 °C for 48 h to determine the hay yield (t ha^−1^). Samples were then ground to pass through a 2-mm screen with a grinder and a subsample was obtained for further analysis. The measurements of plant height (cm) and leaf number per plant were conducted by extending the uppermost fully unfolded leaves using a tape measure in 15 randomly selected plants in each plot.

The crude protein content (%) was calculated by multiplying the total nitrogen (N) concentration by 6.25 using the Kjeldahl method (Association of Analytical Communities (AOAC), 2003). Following an automated fiber analyzer (ANKOM 2000 Fiber Analyser, ANKOM Technology, Macedon, NY, USA), the neutral detergent fiber (NDF; %) and acid detergent fiber (ADF; %) contents were determined following the techniques outlined by Van Soest, Robertson & Lewis (1991). Briefly, 0.5 g of ground sample was put into the fiber filter bag (F57, ANKOM Technology, Macedon, NY, USA) for NDF analysis. The samples were then digested using diluted neutral detergent solution with triethylene glycol (FND20C). For determining ADF, the residues left behind after determining NDF can be directly digested using a solution of acid detergent concentrate and dry CTAB powder (FAD20C, ANKOM Technology, Macedon, NY, USA). Crude protein yield (t ha^−1^) and relative feed value were calculated based on the collected data and the method of Horrocks & Vallentine (1999).

Chlorophyll content (SPAD) was measured using the SPAD-502 device (Konica-Minolta Camera Co. Ltd., Tokyo, Japan) at three phenological stages of sorghum × sudangrass; boot (SPADa), flowering (SPADb), and soft dough (SPADc) stages (Hoel, 1998). Measurements were taken from the topmost fully expanded leaf during vegetative stage, and from the flag leaf during reproductive stages (Rabin & Snapp, 2024). In each plot, SPAD values were recorded from ten randomly selected plants, and the average was used for analysis.

The normalized difference vegetation index (NDVI) values were assessed using a GreenSeeker™ handheld crop sensor (HCS 100, Trimble Ltd., Sunnyvale, CA, USA). Five measurements per plot were taken by holding the sensor 1 m above the crop canopy over a 1 m^2^ area, and the mean value was used for further analysis (Tagarakis et al., 2017).

Leaf area measurements were conducted using a portable leaf area meter (model LI-3000C, LI-COR, Lincoln, NE, USA). For each plot, ten representative plants were sampled at the flowering stage to determine average leaf area. The leaf area index (LAI) was subsequently calculated based on the total leaf area per unit ground area following the standard equation provided by Allamine et al. (2023).

(1) $${\rm LAI} = ({\rm n} \times {\rm LA)}/{\rm A}$$

where LAI is the leaf area index (cm^2^ cm^2^), n is the number of leaves, LA is the mean leaf area (cm^2^), and A is the sampled ground area (cm^2^).

### Soil samples

In order to establish a baseline for soil properties characterization, soil samples were collected from the 0–30 cm layer at three time points: prior to cultivation, after the incorporation of green manures, and following the harvest of sorghum × sudangrass. Samples were taken prior to any treatment application at each phenological stage, allowing a reliable assessment of baseline site conditions. The effect of green manure on soil organic matter content was determined using the Walkley–Black method (Walkley & Black, 1934). Total nitrogen content was analyzed using the Kjeldahl method (Bremner, 1960).

### Statistical analyses

All collected data were subjected to analysis of variance (ANOVA) to determine the effects of treatments on the measured parameters. The experiment included 11 treatments, comprising nine combinations of three forage legume species (common vetch, narbon vetch, and forage pea) and three incorporation stages (pre-flowering, 10% flowering, and 100% flowering), in addition to one fertilized and one unfertilized control. Each treatment was considered a distinct level in a one-way classification design. The experiment followed a randomized complete block design (RCBD) with three replications.

Significant differences among treatment means were assessed using Fisher’s least significant difference (LSD) test at a significance level of *p* < 0.01. LSD values were used to assign letters to treatment groups, where treatments sharing the same letter were not significantly different. These groupings were indicated above error bars in the figures and annotated in all relevant tables.

All analyses were conducted in R software (v.4.2.2; R Core Team, 2022). ANOVA and mean comparisons were performed using the *agricolae* package (De Mendiburu & De Mendiburu, 2019). Pearson correlation analysis was conducted using *metan* package (Olivoto & Lúcio, 2020) to explore relationships among traits. For graphical visualizations, Python programming language was employed with the *Matplotlib* library (Hunter, 2007).

## Results

Green manure applications exhibited statistically significant effects (*p* < 0.01) on all evaluated parameters, including fresh forage yield, hay yield, plant height, leaf number, and forage quality traits such as ADF, NDF, crude protein ratio, crude protein yield, and relative feed value.

The year effect was also significant for LN, ADF, CPR, CPY, chlorophyll content at the booting stage (SPADa), and leaf area index (LAI), reflecting the influence of annual environmental variation on crop performance. Furthermore, the interaction between year and green manure treatments was found to be significant for all parameters except SPAD values, indicating that treatment responses were modulated by year-specific climatic conditions (Table 1).

**Table 1:** Analysis of variance (ANOVA) for sorghum × sudangrass yield components under different green manure treatments.

		Mean squares						
SOV	df	FFY	HY	PH	LN	ADF	NDF	CPR
Year	1	852.48	25.96	1.22	0.66**	21.08**	1.96	1.04*
Green manure app.	10	126,101.82**	3720.34**	3967.2**	13.7**	11.61**	12.13**	6.59**
Year × GM app.	10	768.65**	63.01**	39.09**	0.19*	7.74**	10.88**	0.88**
	**df**	**CPY**	**SPADa**	**SPADb**	**SPADc**	**NDVI**	**LAI**	**RFV**
Year	1	3.33**	14.56**	0.29	0.10	0.0002	0.54*	10.53
Green manure app.	10	51.45**	84.73**	181.06**	159.41**	0.0836**	4.83**	78.37**
Year × GM app.	10	2.91**	3.60	0.39	0.65	0.0029**	0.91**	71.90**

**Note:**

* and ** indicate significance at the 0.05 and 0.01 probability levels, respectively.

### Greeen manure yield

Among the legume species, narbon vetch consistently exhibited the highest green manure biomass across all incorporation stages, reaching 50.60 t ha^−1^ at full flowering. This was followed by forage pea and common vetch, with maximum yields of 44.56 t ha^−1^ and 42.62 t ha^−1^, respectively, also recorded at the full flowering stage. Biomass accumulation increased progressively from the pre-flowering to the full flowering stage in all species, reflecting the typical growth trajectory of maturing legumes. At the pre-flowering stage, yields remained relatively low, ranging between 30.64 and 32.82 t ha^−1^, while intermediate values were observed at 10% flowering, such as 39.40 t ha^−1^ in forage pea and 42.06 t ha^−1^ in narbon vetch. These findings demonstrate that narbon vetch outperformed the other species at all incorporation stages, emphasizing the critical importance of timing in green manure incorporation to optimize biomass production (Table 2).

**Table 2:** Aboveground yields of green manure crops in different phenological stages.

		Aboveground yield (t ha^−1^)
2023		32.37
2024		31.98
***Green manure***	***Phenological stages***	
Common vetch	*Pre-Flowering*	31.35 f
	*%10 Flowering*	39.86 d
	*%100 Flowering*	42.62 c
Narbon vetch	*Pre-Flowering*	32.82 e
	*%10 Flowering*	42.06 c
	*%100 Flowering*	50.60 a
Forage pea	*Pre-Flowering*	30.64 f
	*%10 Flowering*	39.40 d
	*%100 Flowering*	44.56 b
Mean		32.17

**Note:**

The factors indicated by lettering are significant at *p* ≤ 0.05, while the same letters denote non-significant differences.

### Biomass production

The incorporation of different legume species at various phenological stages had a statistically significant impact on the fresh forage yield (FFY) of sorghum × sudangrass. Based on the 2-year average, the highest FFY was observed in the fertilized control treatment, which reached 86.61 t ha^−1^, representing a substantial increase compared to the unfertilized control (32.41 t ha^−1^). Among the green manure treatments, the highest FFY was recorded in common vetch incorporated at the 10% flowering stage (82.03 t ha^−1^), followed closely by narbon vetch incorporated at the pre-flowering stage (78.53 t ha^−1^) and common vetch incorporated at the same stage (77.33 t ha^−1^). Forage pea treatments also exhibited a notable positive influence on FFY, particularly when incorporated at the pre-flowering stage, reaching 76.17 t ha^−1^, markedly outperforming the unfertilized control (Table 3).

**Table 3:** Effects of green manure treatments incorporated in different phenological stages on fresh forage yield (FFY), hay yield (HY), plant height (PH), and leaf number (LN) of sorghum × sudangrass.

		FFY (t ha^−1^)	HY (t ha^−1^)	PH (cm)	LN
2023		70.77	13.65	249.48	10.80 b
2024		71.49	13.53	249.21	11.00 a
***Green manure***	***Phe. stages***				
Control		32.41 I	7.40 ı	176.33 g	6.73 ı
Fertilized		86.61 a	16.81 a	268.66 a	12.91 a
Common vetch	*Pre-Flo*	77.33 cd	13.41 ef	265.00 ab	11.53 b
	*10% Flo*	82.03 b	15.55 b	264.83 ab	11.51 bc
	*100% Flo*	73.57 f	12.68 g	252.00 de	11.00 df
Narbon vetch	*Pre-Flo*	78.53 c	14.94 cd	254.33 cd	11.51 bc
	*10% Flo*	74.34 ef	13.16 fg	251.83 de	11.18 ce
	*100% Flo*	60.74 h	11.97 h	239.16 f	10.60 g
Forage pea	*Pre-Flo*	76.17 de	15.39 bc	263.50 b	11.31 bd
	*10% Flo*	70.49 g	14.49 d	258.33 c	10.88 eg
	*100% Flo*	70.21 g	13.69 e	248.83 e	10.76 fg
Mean		71.13	13.59	249.34	10.90

**Note:**

The factors indicated by lettering are significant at *p* ≤ 0.05, while the same letters denote non-significant differences.

**Figure 3:**
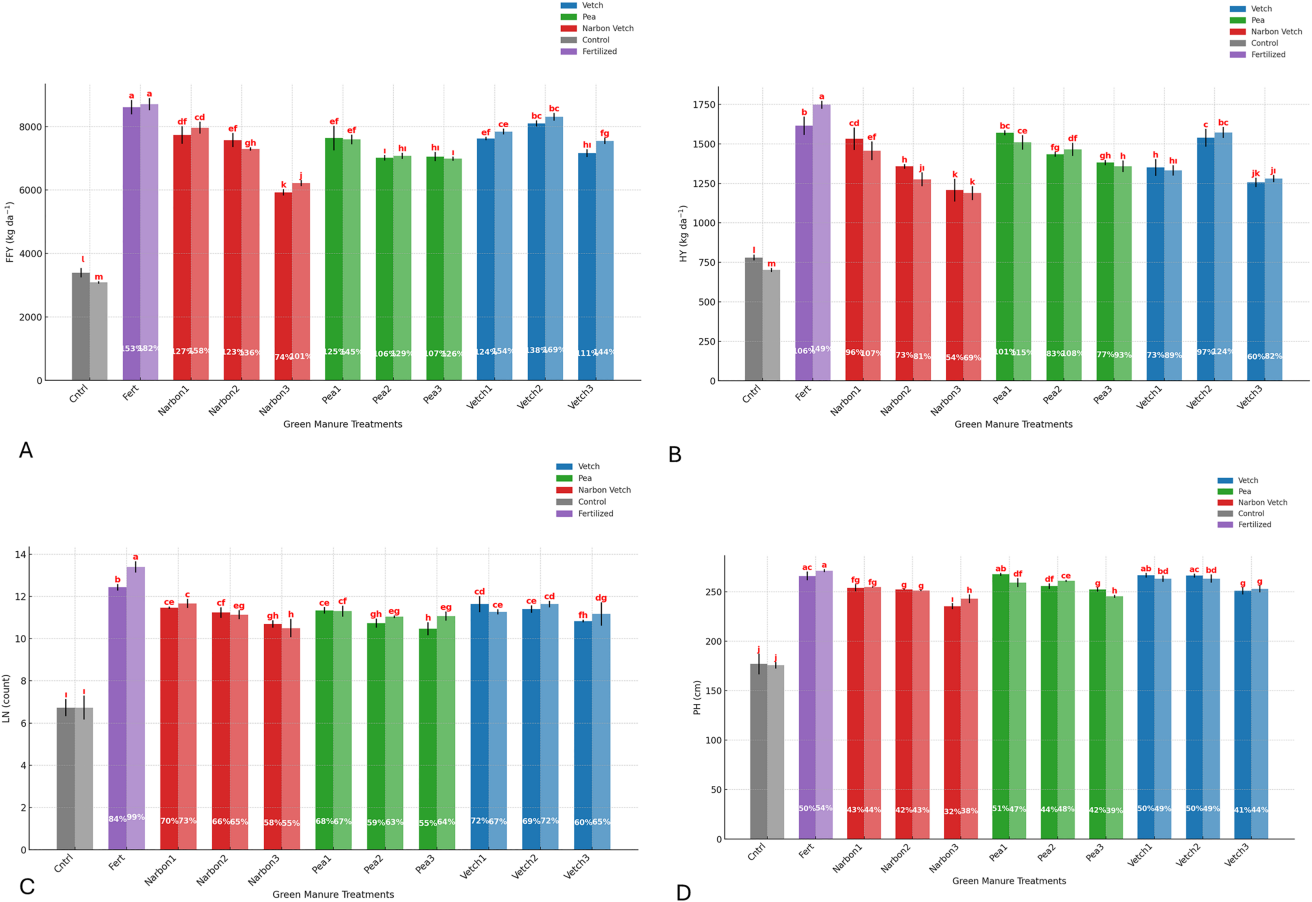
Effect of different green manure treatments on yield and growth parameters. (A) Fresh forage yield (FFY, kg da^−1^); (B) hay yield (HY, kg da^−1^); (C) plant height (PH, cm); (D) leaf number per plant (LN, count). For each green manure treatment the first bar represents values from 2023, while the second bar represents values from 2024).

The interaction between year and green manure treatments further highlighted the role of climatic variation. In the second year, the fertilized control outperformed the unfertilized control by 182%, while common vetch treatments showed yield increases of up to 169%. In contrast, narbon vetch at full flowering resulted in the lowest relative increase, with yields approximately 74% higher than the unfertilized control (Fig. 3).

Hay yield (HY) varied markedly depending on the legume species and the phenological stage at which green manure was incorporated into the soil. The highest HY was observed in the fertilized control, reaching 16.81 t ha^−1^, while the lowest yield was recorded in the unfertilized control (7.40 t ha^−1^). Among green manure treatments, common vetch incorporated at the 10% flowering stage produced the highest HY (15.55 t ha^−1^), closely followed by forage pea incorporated at the pre-flowering stage, which achieved 15.39 t ha^−1^. These findings highlight the superior biomass contribution of certain species when incorporated at optimal developmental stages. In general, green manure applications led to statistically significant improvements in HY compared to the unfertilized control. However, delayed incorporation (*e.g*., at full flowering) tended to be less effective, suggesting that early-stage incorporation is more favorable for maximizing sorghum × sudangrass productivity. A trend consistent with fresh forage yield was also observed across species, reinforcing the role of phenological timing. Notably, a strong interaction between year and treatment was identified: common vetch incorporated at 10% flowering led to a remarkable 124% increase in HY over the unfertilized control in the second year. Nevertheless, the greatest overall enhancement was recorded in the fertilized control, which yielded a 149% increase relative to the baseline (Table 3; Fig. 3).

Plant height was significantly influenced by green manure applications, with notable differences observed across legume species and their respective incorporation stages. Based on the 2-year average, the tallest plants were recorded under the fertilized control treatment (268.66 cm), closely followed by common vetch incorporated at the pre-flowering (265.00 cm) and 10% flowering (264.83 cm) stages, both of which were statistically comparable to the fertilized control. The shortest plants were observed in the unfertilized control, where sorghum × sudangrass reached only 176.33 cm. Among green manure treatments, narbon vetch resulted in the lowest plant height improvements relative to the unfertilized control. Notably, the interaction between year and treatment revealed that plant heights under the fertilized control and under common vetch applied at both the pre-flowering and 10% flowering stages were comparable. In the second year of the experiment, the fertilized control exhibited the greatest relative increase in plant height over the unfertilized control at 54%. This was closely followed by common vetch applied at the pre-flowering and 10% flowering stages, which achieved 50% and 49% increases, respectively. These findings highlight the effectiveness of early-stage common vetch incorporation in promoting vegetative growth in sorghum × sudangrass, nearly matching the performance of mineral fertilization (Table 3; Fig. 3).

Leaf number per plant was significantly influenced by the interaction between green manure treatments and experimental years. Although slight year-to-year variations were observed, leaf counts were generally higher in 2023 compared to 2022, suggesting more favorable climatic conditions during the second year. Based on the 2-year average, the highest leaf number (12.91 leaves per plant) was recorded under the fertilized control, reaffirming the positive contribution of mineral fertilization to vegetative development. Among the green manure treatments, the incorporation of all three legume species at the pre-flowering stage and common vetch at the 10% flowering stage yielded the greatest leaf numbers in sorghum × sudangrass. In contrast, the lowest leaf count (6.73 leaves per plant) was observed in the unfertilized control, reflecting the limiting effect of nutrient deficiency on foliar development. These findings are consistent with previously reported trends in fresh and dry forage yield and plant height, underscoring once again the critical role of appropriate legume species selection and incorporation timing in optimizing sorghum × sudangrass performance within green manure-based nutrient management systems (Table 3; Fig. 3).

### Forage quality

ADF content was significantly influenced by year, green manure treatments, and their interaction (*p* < 0.01). Among all treatments, the highest ADF value was recorded in the fertilized plots (39.08%), representing an increase compared to the unfertilized control (36.56%). Among the legume-based green manure treatments, forage pea incorporated at the pre-flowering stage exhibited the highest ADF content (38.96%), which was higher than the control and closely approximated the value observed in the fertilized treatment. Conversely, the lowest ADF level was observed in the common vetch treatment incorporated at the pre-flowering stage (34.58%), indicating a notable reduction relative to the unfertilized control. Incorporation timing exerted a clear influence; particularly, pre-flowering applications of common vetch and full-flowering applications of narbon vetch resulted in lower ADF concentrations, suggesting enhanced forage digestibility. According to the interaction effect, ADF contents generally increased in the second year; however, this trend diverged in the case of common vetch, indicating a species-specific response to interannual variability (Table 4).

**Table 4:** Effects of green manure treatments incorporated in different phenological stages on ADF, NDF, crude protein ratio (CPR), crude protein yield (CPY) of sorghum × sudangrass.

		ADF (%)	NDF (%)	CPR (%)	CPY (t ha^−1^)
2023		36.48 b	56.95	9.97 a	1.372 a
2024		37.61 a	57.30	9.72 b	1.327 b
***Green manure***	***Phe. stages***				
Control		36.56 de	59.40 a	7.32 h	0.543 f
Fertilized		39.08 a	58.01 b	9.79 ef	0.164 a
Common vetch	*Pre-Flo*	34.58 f	55.55 d	11.28 a	1.513 bc
	*%10 Flo*	37.80 bc	57.43 bc	9.61 eg	1.496 bc
	*%100 Flo*	36.51 de	55.91 d	10.04 de	1.274 e
Narbon vetch	*Pre-Flo*	36.98 cd	56.33 cd	10.41 cd	1.556 b
	*%10 Flo*	36.21 de	55.65 d	10.57 bc	1.391 d
	*%100 Flo*	35.68 e	55.28 d	10.89 ab	1.304 e
Forage Pea	*Pre-Flo*	38.96 a	58.26 ab	9.39 fg	1.446 cd
	*%10 Flo*	38.36 ab	58.08 b	9.69 eg	1.404 d
	*%100 Flo*	36.75 d	58.46 ab	9.30 g	1.275 e
Mean		37.04	57.12	9.84	1.350

**Note:**

The factors indicated by lettering are significant at *p* ≤ 0.05, while the same letters denote non-significant differences.

**Figure 4:**
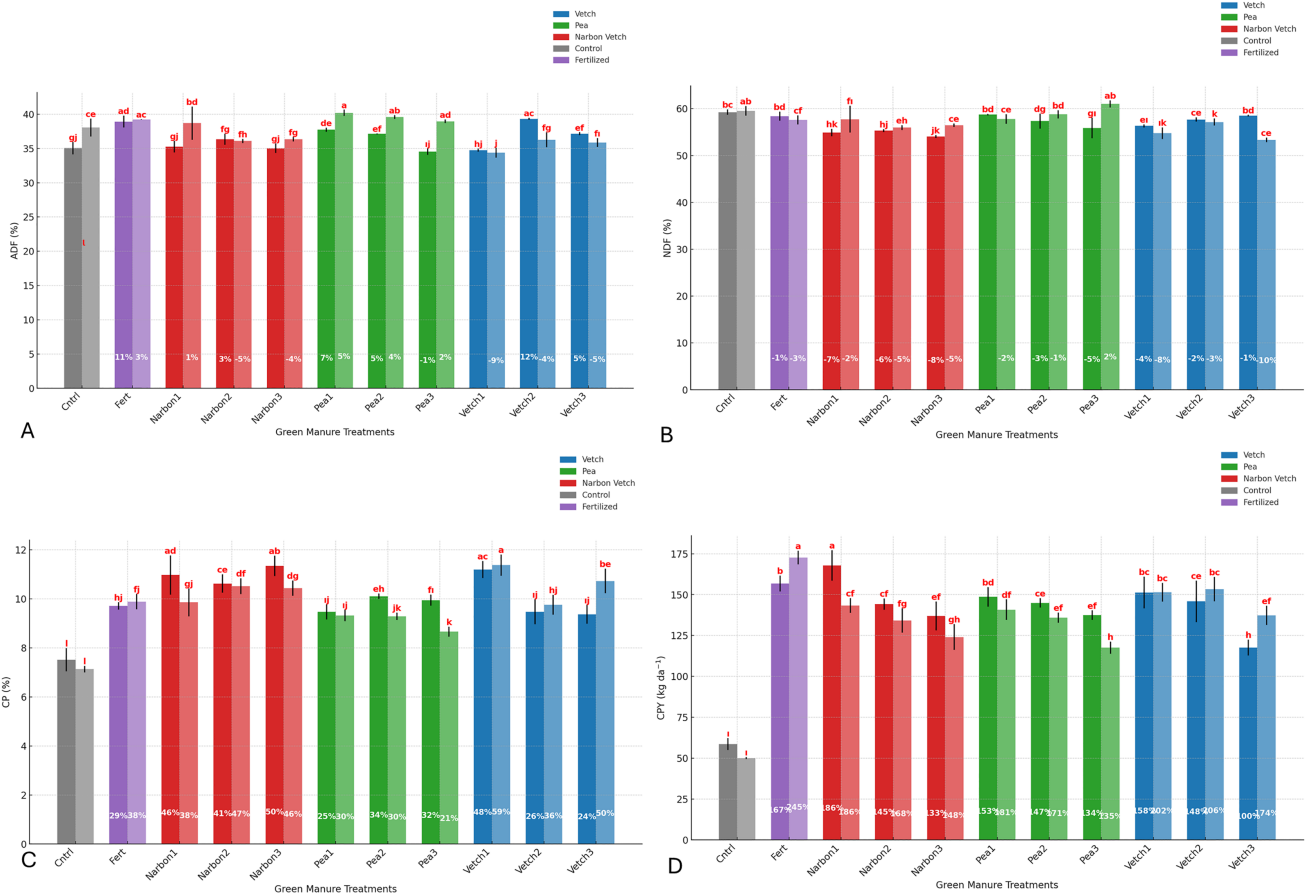
Effect of different green manure treatments on yield and growth parameters. (A) ADF (%) (B) NDF (%); (C) crude protein ratio; (D) crude protein yield. For each green manure treatment, the first bar: 2023, second bar: 2024).

Unlike ADF, NDF content was not significantly affected by year. However, green manure species and incorporation timing caused notable variations. The highest NDF value was observed in the unfertilized control (59.40%), while lower values were recorded for common vetch at pre-flowering and 100% flowering, and for narbon vetch at 10% and 100% flowering stages. These reductions indicate improved forage quality due to lower fiber concentration. Among legume treatments, common vetch and narbon vetch generally resulted in lower NDF levels compared to other species. In contrast, forage pea maintained relatively high NDF contents across all incorporation stages, with the highest value (58.46%) observed at 100% flowering. According to the year × treatment interaction, forage pea incorporated at full flowering in the second year showed the highest NDF content among green manure treatments, closely resembling the unfertilized control. These results emphasize the importance of legume selection and incorporation timing in managing forage fiber composition (Table 4; Fig. 4).

The crude protein ratio and crude protein yield of sorghum × sudangrass were significantly influenced by green manure species, incorporation timing, and growing season (*p* < 0.01). Both parameters exhibited superior outcomes in 2023 compared to 2024, likely due to more favorable climatic conditions that enhanced nitrogen mineralization and uptake efficiency. Among all treatments, the highest CPR was recorded for common vetch incorporated at the pre-flowering stage (11.28%), followed by narbon vetch at 100% flowering (10.89%) and 10% flowering (10.57%). These values were substantially higher than that of the unfertilized control (7.32%), highlighting the protein-enhancing potential of early-stage legume incorporation. According to the year × treatment interaction, all green manure treatments, except forage pea, generally outperformed the fertilized control in CPR. The most pronounced CPR increase over the unfertilized control was observed under pre-flowering narbon vetch in the second year, reaching 59%, followed by 100% and pre-flowering narbon vetch in the first year (Table 4; Fig. 4).

A consistent response pattern was also evident in CPY, with the highest value achieved under the fertilized control (1.64 t ha^−1^). Among legume-based treatments, narbon vetch incorporated at the pre-flowering stage yielded 1.55 t ha^−1^, followed by common vetch at pre-flowering (1.51 t ha^−1^) and 10% flowering (1.49 t ha^−1^). Notably, the year × treatment interaction revealed that narbon vetch at the pre-flowering stage in the first year led to the most substantial improvement, producing a CPY 186% higher than the unfertilized control—surpassing even the fertilized treatment. In contrast, forage pea showed comparatively lower CPR and CPY values across all stages, particularly at full flowering. Nonetheless, even forage pea applications yielded 134% to 171% higher CPY than the unfertilized control, underscoring the general effectiveness of legume green manure strategies (Table 4; Fig. 4).

### Physiological traits

**Figure 5:**
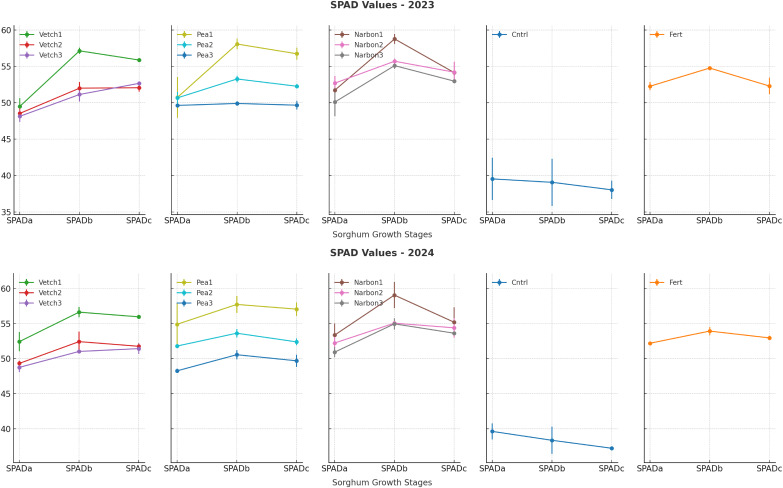
SPAD values of sorghum × sudangrass at different growth stages under various green manure treatments (SPADa: boot SPADb: flowering SPADc: soft dough). Error bars represent the standard error of the mean (SE).

The chlorophyll content of sorghum × sudangrass, assessed *via* SPAD readings at three phenological stages (SPADa—boot, SPADb—flowering, SPADc—soft dough), was significantly influenced by green manure species and incorporation timing (*p* < 0.01). Across all growth stages, early-stage green manure incorporation, particularly at the pre-flowering stage, consistently yielded the highest SPAD values. At the boot stage (SPADa), chlorophyll levels were markedly elevated in plots where narbon vetch (52.55) and forage pea (52.81) were incorporated at the pre-flowering stage. Similarly, at flowering (SPADb), early incorporation again led to superior SPAD values, with narbon vetch (58.91), forage pea (57.90), and common vetch (56.88) outperforming later treatments. During the soft dough stage (SPADc), pre-flowering incorporation maintained its advantage, with forage pea reaching 56.90 and common vetch 55.91 (Table 5; Fig. 5).

**Table 5:** Effects of green manure treatments incorporated in different phenological stages on SPAD and NDVI of sorghum × sudangrass.

		SPADa	SPADb	SPADc	NDVI
2023		49.41 b	53.17	51.90	0.786
2024		50.35 a	53.03	51.97	0.782
***Green manure***	***Phe. stages***				
Control		39.58 f	38.71 h	37.63 g	0.476 f
Fertilized		52.23 ab	54.35 cd	52.63 de	0.908 a
Common vetch	*Pre-Flo*	50.96 bc	56.88 b	55.91 a	0.826 c
	*10% Flo*	48.93 de	52.21 ef	51.91 e	0.736 e
	*100% Flo*	48.45 e	51.08 fg	52.05 e	0.720 e
Narbon vetch	*Pre-Flo*	52.55 ab	58.91 a	54.66 b	0.850 bc
	*10% Flo*	52.46 ab	55.38 c	54.30 bc	0.855 bc
	*100% Flo*	50.51 cd	55.03 c	53.30 cd	0.773 d
Forage Pea	*Pre-Flo*	52.81 a	57.90 ab	56.90 a	0.871 b
	*10% Flo*	51.23 ac	53.45 de	52.33 de	0.846 bc
	*100% Flo*	48.95 de	50.23 g	49.68 f	0.766 d
Mean		49.88	53.10	51.93	0.784

**Note:**

SPADa: Boot time SPAD, SPADb: Flowering SPAD, SPADc: Soft dough SPAD. The factors indicated by lettering are significant at *p* ≤ 0.05, while the same letters denote non-significant differences.

In contrast, incorporation at full flowering (100%) consistently produced the lowest chlorophyll readings for instance, forage pea dropped to 49.68. At all three measurement points, control plots exhibited the lowest chlorophyll content, with values progressively declining as the crop advanced through its phenological stages. Fertilized plots performed moderately better than the control, but generally failed to match the chlorophyll levels observed in early green manure applications. These findings underscore that green manure incorporation, especially at the pre-flowering stage, can be equally or even more effective than conventional fertilization in maintaining chlorophyll content and enhancing nitrogen availability during key stages of plant development. As such, early legume-based green manure application emerges as a viable and sustainable alternative to mineral fertilization strategies for optimizing sorghum × sudangrass physiological performance (Table 5; Fig. 5).

NDVI of sorghum × sudangrass was significantly affected by green manure species and their incorporation timing (*p* < 0.01), whereas the year effect was not statistically significant. The highest NDVI value (0.908) was recorded in the fertilized control, demonstrating the positive impact of mineral fertilization on canopy development. However, green manure treatments also markedly enhanced NDVI relative to the unfertilized control (0.476), underscoring their contribution to vegetative vigor. Among the legume-based treatments, forage pea incorporated at the pre-flowering (0.871) and 10% flowering (0.846) stages, as well as narbon vetch applied at pre-flowering (0.850) and 10% flowering (0.855), achieved the highest NDVI values across both years. In contrast, full-flowering incorporation generally resulted in diminished NDVI values, indicating reduced canopy performance when application timing was delayed.

**Figure 6:**
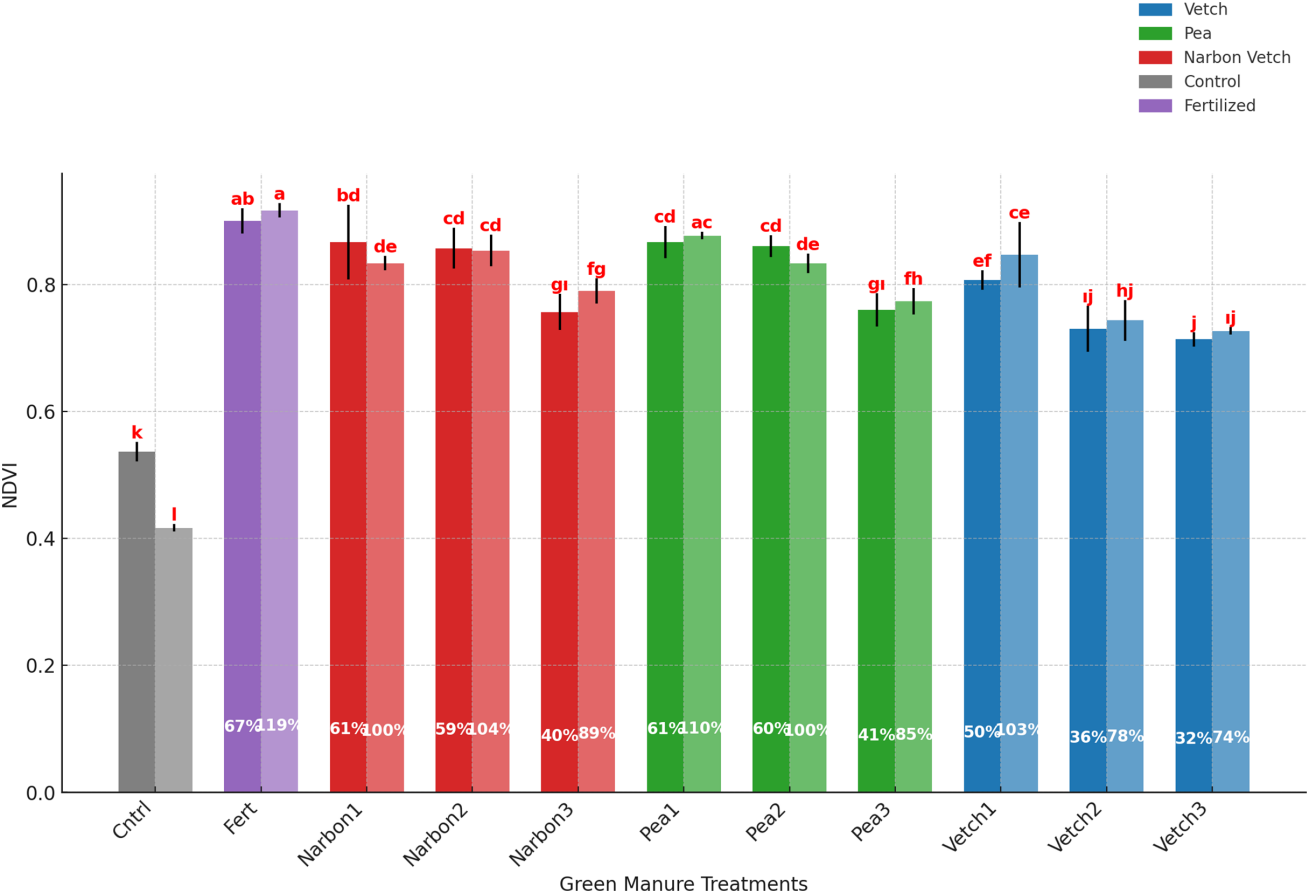
Effects of green manure treatments incorporated in different phenological stages on SPAD and NDVI of Sorghum × sudangrass. The factors indicated by lettering are significant at *p* ≤ 0.05, while the same letters denote non-significant differences.

Interaction effects revealed interannual variability in response to legume type and incorporation stage. Nevertheless, a consistent trend emerged: the contribution of green manure to NDVI tended to decline as phenological development progressed. The fertilized control remained superior in both years, showing 67% and 119% increases over the unfertilized control in the first and second years, respectively. Among green manure treatments, the most pronounced enhancement was observed with forage pea incorporated at the pre-flowering stage in the second year, exhibiting a 110% NDVI increase relative to the unfertilized control. Conversely, the smallest gain was recorded under common vetch incorporated at full flowering in the first year, which still provided a 32% improvement (Table 5; Fig. 6).

**Figure 7:**
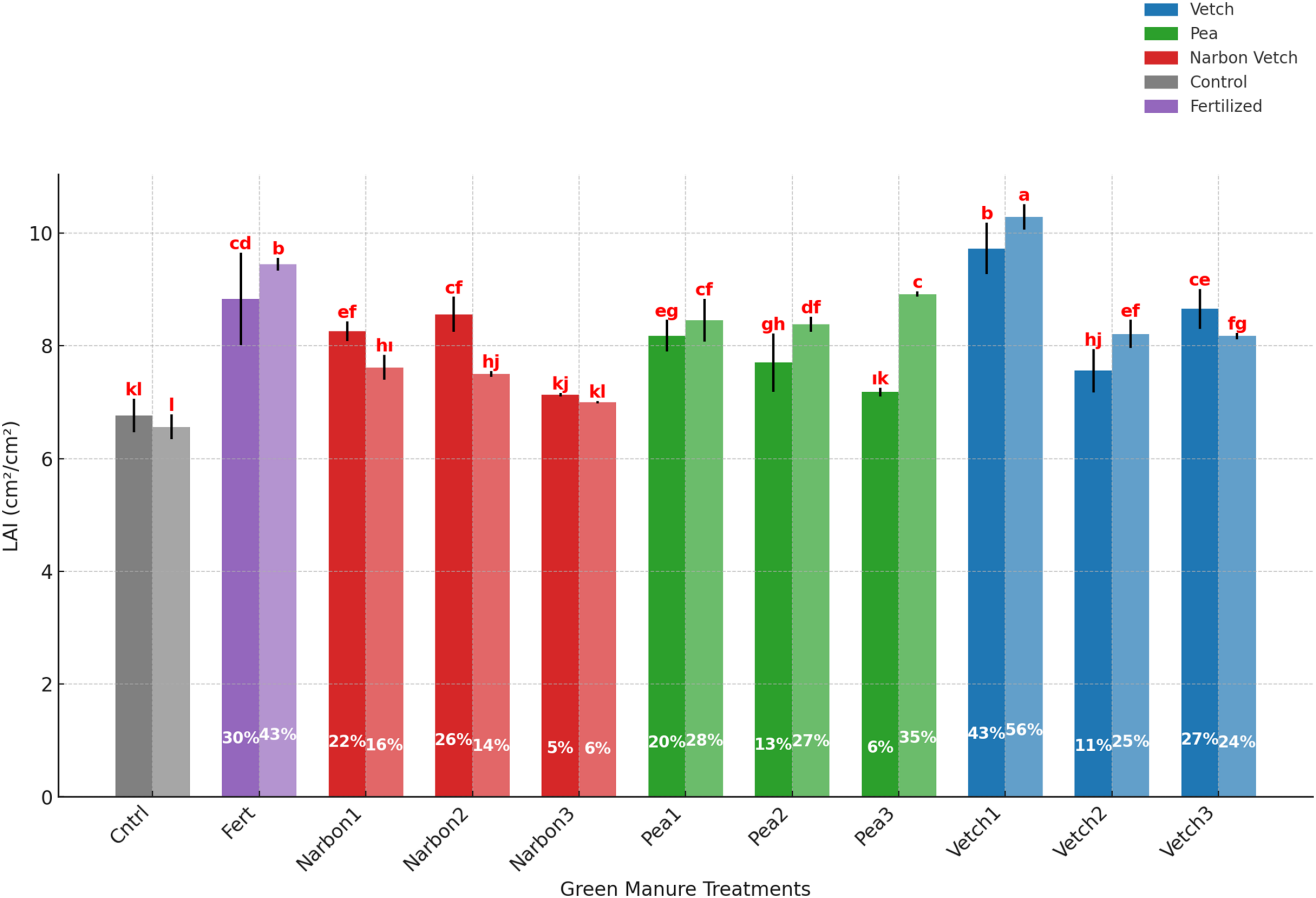
Effect of different green manure treatments on LAI in 2023 and 2024. (For each green manure treatment, the first bar represents values from 2023, while the second bar represents values from 2024). Error bars represent the standard error of the mean (SE).

According to the results, the leaf area index (LAI) of sorghum × sudangrass was significantly influenced by the green manure species, incorporation timing, and year. Green manure applications conducted at early phenological stages, particularly with common vetch and forage pea, resulted in higher LAI values compared to later-stage applications and the unfertilized control. However, narbon vetch exhibited a distinct pattern, differing from the other species. Based on the 2-year average, common vetch incorporated at the pre-flowering stage produced the highest LAI (10.01 cm^2^ cm^−2^), clearly outperforming all other green manure species and incorporation timings. This treatment enhanced LAI by 43% in the first year and 56% in the second year relative to the unfertilized control. Similarly, common vetch incorporated at 100% flowering (8.41 cm^2^ cm^−2^), and forage pea applied at pre-flowering (8.31 cm^2^ cm^−2^), also led to notable improvements over the control. In contrast, narbon vetch consistently produced lower LAI values across all incorporation timings. The lowest LAI (7.07 cm^2^ cm^−2^) was recorded under narbon vetch applied at full flowering, with only modest increases of 5% and 6% over the control in the first and second years, respectively. While the fertilized control achieved a higher LAI (9.14 cm^2^ cm^−2^) than the unfertilized control, it remained lower than the value obtained from early incorporation of common vetch These findings highlight the superior potential of early-incorporated common vetch in promoting leaf area development, demonstrating performance that exceeds not only the unfertilized control but also the mineral fertilization strategy (Table 6; Fig. 7).

**Table 6:** Effects of green manure treatments incorporated in different phenological stages on LAI of sorghum × sudangrass.

		LAI (cm^2^ cm^−2^)
2023		8.05 b
2024		8.23 a
***Green manure***	***Phenological stages***	
Control		6.66 g
Fertilized		9.14 b
Common vetch	*Pre-Flowering*	10.01 a
	*%10 Flowering*	7.88 e
	*%100 Flowering*	8.41 c
Narbon vetch	*Pre-Flowering*	7.94 e
	*%10 Flowering*	8.03 de
	*%100 Flowering*	7.07 f
Forage pea	*Pre-Flowering*	8.31 cd
	*%10 Flowering*	8.04 de
	*%100 Flowering*	8.05 de
Mean		8.14

**Note:**

The factors indicated by lettering are significant at *p* ≤ 0.05, while the same letters denote non-significant differences.

### Relative feed value

**Figure 8:**
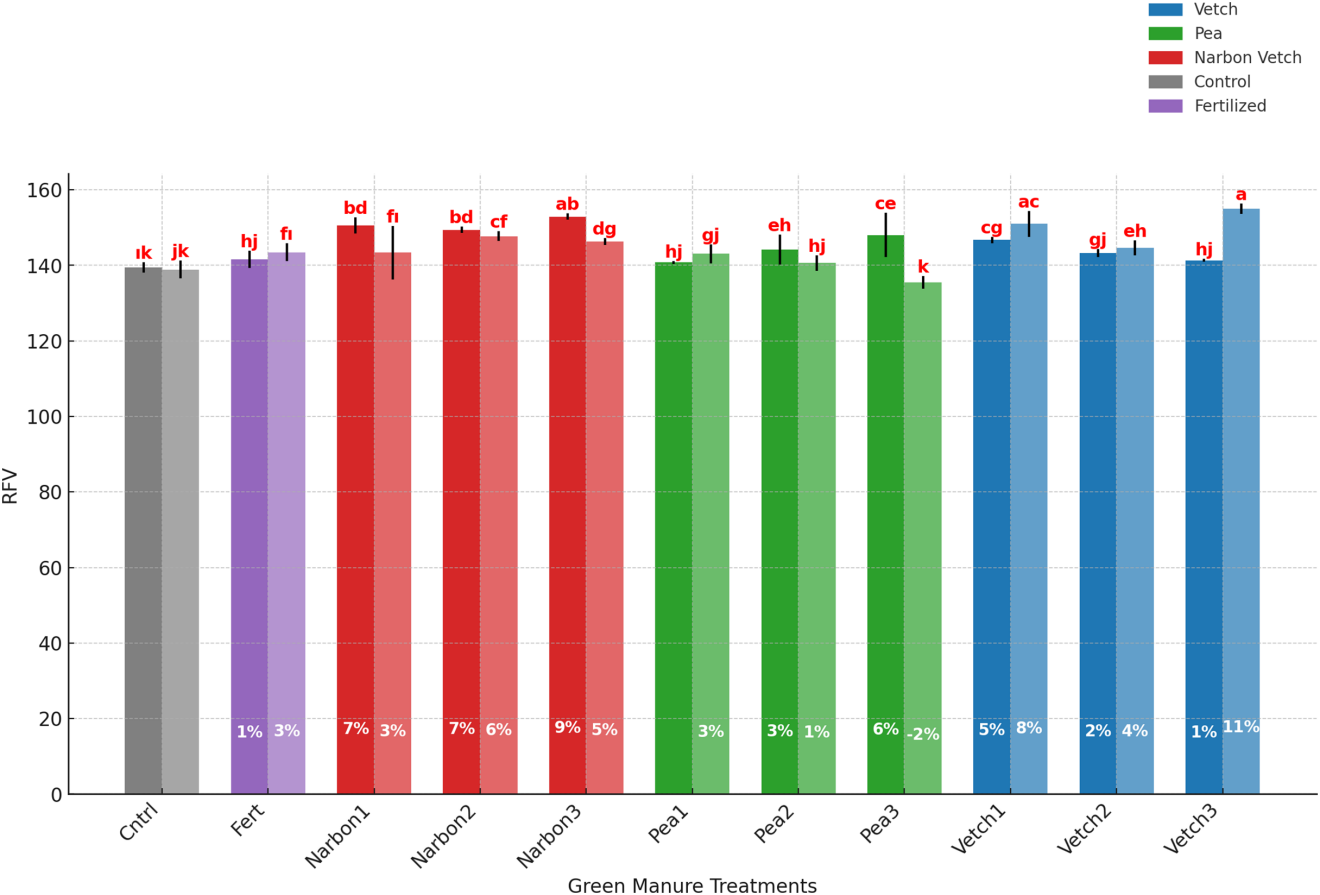
Effect of different green manure treatments on RFV in 2023 and 2024 (For each green manure treatment, the first bar represents values from 2023, while the second bar represents values from 2024). Error bars represent the standard error of the mean (SE).

The relative feed value of sorghum × sudangrass was significantly influenced by both the green manure species and their incorporation timing. The highest RFV values were recorded in treatments involving narbon vetch and common vetch, with the latter consistently achieving statistically superior values across all incorporation stages except at 10% flowering. In these treatments, RFV ranged from 146.98 to 149.62, with the highest value observed in narbon vetch incorporated at the full flowering stage (149.62). In contrast, forage pea treatments resulted in comparatively lower RFV values, and no statistically significant differences were detected among its incorporation stages (ranging between 141.76 and 142.41). All green manure applications led to improvements in RFV compared to the unfertilized control plots. Notably, narbon vetch applied at full flowering enhanced RFV by approximately 9% in the first year. A particularly noteworthy finding is that the fertilized control failed to surpass the RFV levels attained by the most effective green manure treatments. These results underscore the potential of specific legume species—particularly narbon vetch and common vetch—to enhance forage quality when incorporated at appropriate phenological stages, offering a viable alternative to mineral fertilization (Table 7; Fig. 8).

**Table 7:** Effects of green manure treatments incorporated in different phenological stages on relative feed value sorghum × sudangrass.

		RFV
2023		145.31
2024		144.51
***Green manure***	***Phenological stages***	
Control		139.20 d
Fertilized		142.54 c
Common vetch	*Pre-Flowering*	148.89 a
	*10% Flowering*	143.97 bc
	*100% Flowering*	148.18 a
Narbon vetch	*Pre-Flowering*	146.98 ab
	*10% Flowering*	148.57 a
	*100% Flowering*	149.62 a
Forage pea	*Pre-Flowering*	141.92 cd
	*10% Flowering*	142.41 c
	*100% Flowering*	141.76 cd
Mean		144.91

**Note:**

The factors indicated by lettering are significant at *p* ≤ 0.05, while the same letters denote non-significant differences.

### Pearson correlation’s of phenological stages of green manures applied to sorghum-sudangrass

**Figure 9:**
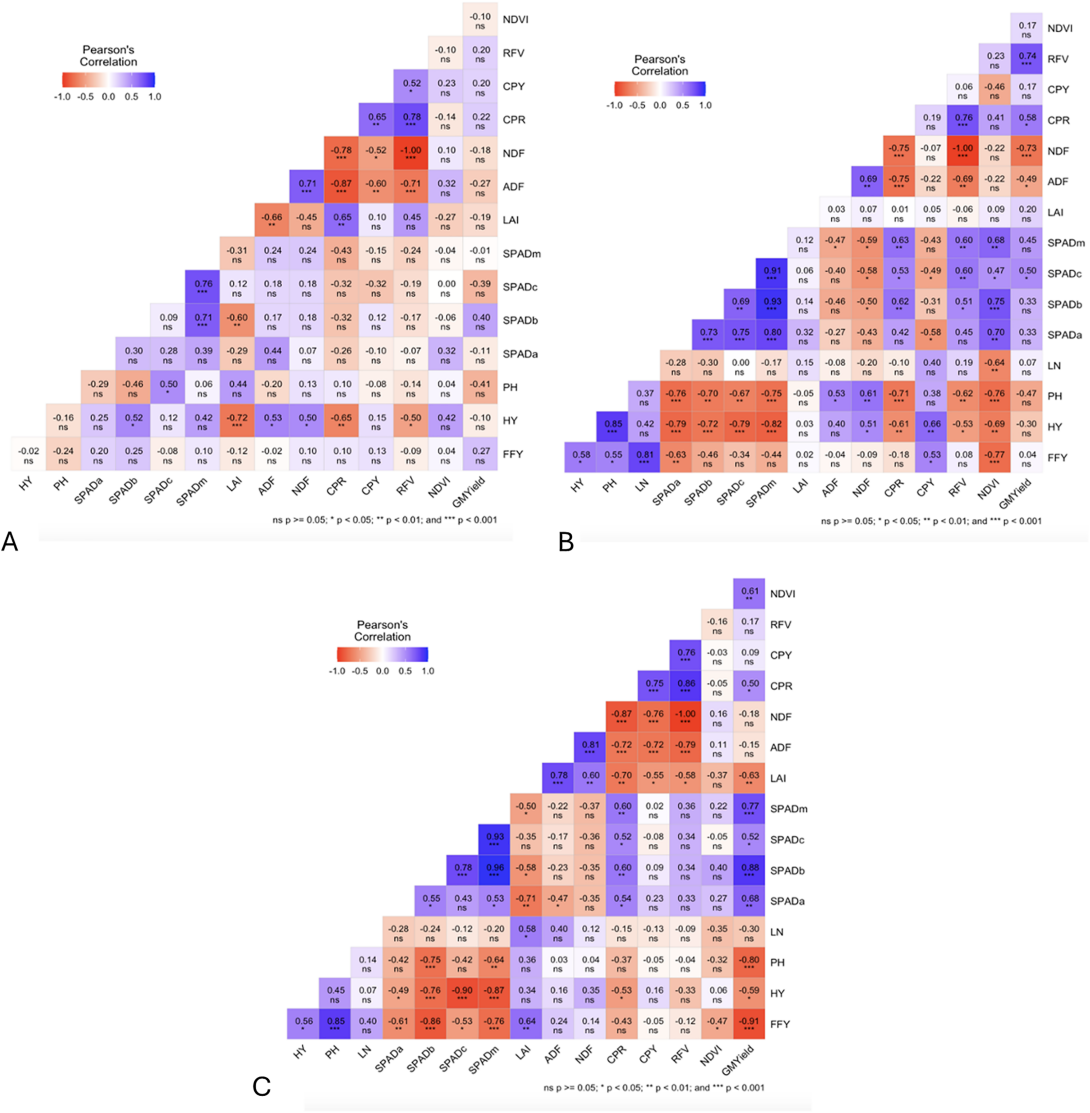
Correlograms of different phenological stages (A) pre-flowering, (B) 10% flowering, (C) 100% flowering) of green manure treatments of sorghum-sudangrass production. (HY, hay yield; PH, plant height; LN, leaf number per plant; SPADa: ; SPADb: ; SPADc: ; LAI, leaf area index; ADF, aicd detergent fiber; NDF, neutral detergent fiber; CPR, crude protein ratio; CPY, crude protein yield; RFV, relative feed value).

At the pre-flowering stage, several statistically significant correlations were identified among yield, physiological, and forage quality parameters. A particularly strong negative correlation was observed between relative feed value (RFV) and neutral detergent fiber (NDF) (r = −1.00), reinforcing the well-established inverse relationship between fiber content and forage quality. Likewise, acid detergent fiber (ADF) and NDF showed a strong negative correlation (r = −0.87), reflecting similar behavior in fiber fractions. Hay yield (HY) was negatively correlated with both the leaf area index (LAI; r = −0.72) and crude protein ratio (CPR; r = −0.65), suggesting that early-stage biomass accumulation may limit either canopy expansion or nitrogen assimilation (Fig. 9A).

In contrast, RFV was positively correlated with CPR (r = 0.78), confirming the role of protein concentration in enhancing forage quality. A moderate positive correlation between LAI and CPR (r = 0.65) indicates that canopy development contributes to nitrogen uptake. Similarly, CPR and crude protein yield (CPY) were positively correlated (r = 0.65), reflecting synchronized improvements in both protein concentration and total nitrogen accumulation as a result of early green manure incorporation (Fig. 9A).

At the 10% flowering incorporation stage, the correlation patterns became more complex, reflecting physiological transitions in plant development. A strong positive correlation was recorded between leaf number (LN) and fresh forage yield (FFY) (r = 0.81), as well as between plant height (PH) and hay yield (HY) (r = 0.85), highlighting the importance of vegetative growth in biomass production. CPY also showed positive associations with HY (r = 0.66), and NDF was positively linked to PH (r = 0.61). Additionally, NDVI exhibited positive correlations with all SPAD readings, while CPR and RFV were positively associated with all SPAD indices except SPADa, emphasizing the relationship between chlorophyll content and nitrogen-based quality traits (Fig. 9B).

Nevertheless, several notable negative correlations emerged. SPADa showed a negative association with FFY (r = –0.63), while NDVI was also negatively correlated with FFY (r = −0.77). All SPAD readings were negatively correlated with both HY and PH, suggesting potential trade-offs between chlorophyll concentration and structural biomass. Additionally, CPR and HY (r = −0.61) and NDVI and HY (r = −0.69) showed inverse relationships. Strong negative correlations were also observed between CPR and ADF/NDF, and between RFV and NDF (r = −1.00), reaffirming the negative impact of fiber accumulation on forage quality (Fig. 9B).

Under the full flowering incorporation stage, correlation structures remained robust (Fig. 9C). A strong positive relationship was found between PH and FFY (r = 0.85), and a moderate positive correlation between LAI and FFY (r = 0.64), indicating that canopy size continues to influence biomass accumulation. ADF and LAI were positively correlated (r = 0.78), as were NDF and ADF (r = 0.81), suggesting coordinated increases in fiber components with plant maturity. Importantly, RFV was strongly and positively associated with both CPR (r = 0.86) and CPY (r = 0.76), further validating the relevance of protein content in determining feed quality.

Conversely, all SPAD indices were negatively correlated with FFY and HY, and SPADb showed a negative relationship with PH, indicating possible declines in chlorophyll content under delayed incorporation. LAI was negatively associated with SPADa, hinting at reduced canopy light penetration or altered leaf structure at later stages. Notably, CPR, CPY, and RFV all showed strong negative correlations with both ADF and NDF, emphasizing the adverse effects of fiber accumulation on forage digestibility and nutritional value. Furthermore, green manure biomass yield was negatively correlated with FFY, suggesting that higher green manure productivity may not always translate into enhanced subsequent crop yields under late incorporation (Fig. 9C).

### Soil organic matter and total nitrogen

**Figure 10:**
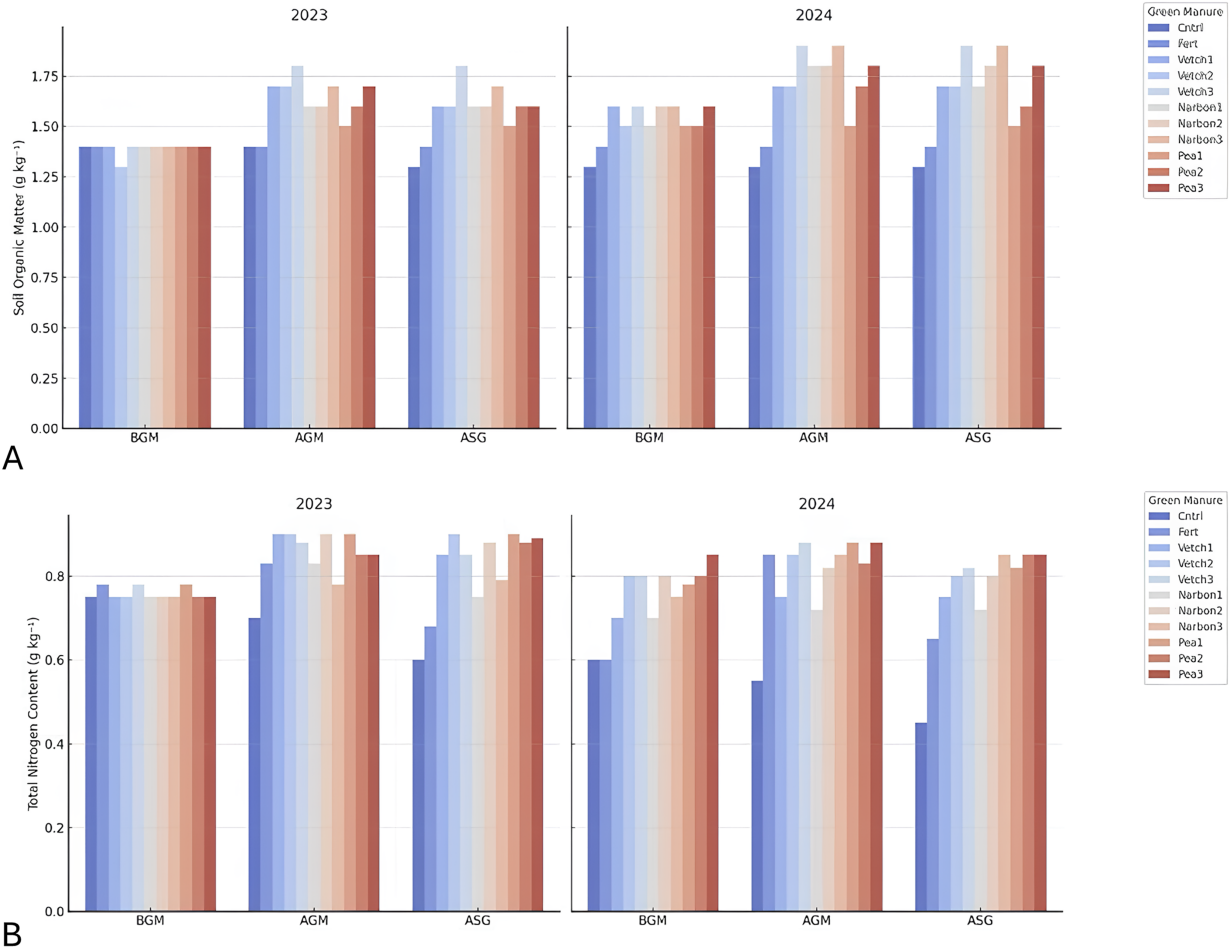
Soil characteristics of green manure-Sorghum × sudangrass production. BGM, before green manure treatment, AGM, after green manure treatment, ASG, after sorghum harvest.

Soil organic matter (SOM) content exhibited a marked improvement following the incorporation of legume-based green manures. Initially present at low levels prior to incorporation, SOM increased substantially after application, frequently exceeding 1.50 g kg^−1^, particularly under treatments with common vetch, narbon vetch, and forage pea. Although a slight decline was observed after the sorghum harvest, SOM levels remained notably higher than baseline values, indicating that green manure incorporation contributed to the buildup of soil organic carbon while partially offsetting the depletion caused by the subsequent cropping cycle. Moreover, SOM levels in 2024 were marginally higher than in 2023, suggesting a cumulative benefit of repeated green manure applications on long-term soil health. In terms of incorporation timing, applications made between 10% and 100% flowering stages were especially effective in enhancing SOM levels, underscoring the critical role of phenological stage in maximizing organic matter contribution (Fig. 10A). Total nitrogen (TN) content in the soil followed a similar trajectory. The lowest TN levels were recorded prior to green manure incorporation, followed by significant increases across legume-based treatments. In several instances, TN concentrations reached or surpassed 0.80 g kg^−1^, highlighting the capacity of these species to enhance soil nitrogen status. Despite minor reductions following sorghum harvest, TN levels remained well above initial values. The slightly elevated TN content observed in 2024 further reinforces the sustained impact of green manures on nitrogen enrichment over time. Although there was some variability in the response across incorporation timings, applications made at approximately 10% flowering—particularly with common vetch—proved most effective in preserving soil nitrogen levels (Fig. 10B).

## Discussion

The increasing environmental concerns and the high cost of mineral fertilizers have underscored the significance of alternative approaches in agricultural production. In this context, legume-based green manure applications emerge as a promising strategy for sustainable agriculture by enhancing soil health through biological nitrogen fixation, increased enzymatic activity, and organic matter accumulation (Anas et al., 2020; De Vries, 2021; Ács et al., 2024; Asghar & Kataoka, 2021). Beyond short-term yield improvements, green manuring also exerts long-term positive effects on soil structure, moisture retention, and nutrient cycling (Joshna & Bokado, 2024; Shao et al., 2024).

This study demonstrated that the type of green manure applied, and the timing of its incorporation significantly influenced the yield, quality, and physiological development of sorghum × sudangrass grown under Mediterranean climate conditions. All green manure treatments resulted in significant improvements compared to the control plots. Notably, early and pre-flowering incorporation of common vetch showed superior performance in terms of both yield and forage quality. These findings underscore the critical role of both phenological timing and species selection in maximizing the effectiveness of green manure applications.

Common vetch is distinguished by its atmospheric nitrogen fixation capacity, low C:N ratio, and rapid decomposability (Parissi et al., 2022; Ribeiro et al., 2022). These characteristics enable synchronized nitrogen release during the crop growth period, leading to enhanced physiological parameters such as SPAD, LAI, and NDVI (Akbar et al., 2024; Han et al., 2021). Moreover, its high biomass production and contribution to soil organic matter improve soil structure (Nguyen et al., 2020; Çağlar, Aydemir & Kaçan, 2025). Although early incorporation may appear to have lower immediate effects compared to later stages, its impact on the subsequent crop can be limited due to earlier decomposition. Similarly, forage pea applied in early stages enhanced soil nitrogen and microbial activity, thereby supporting crop development (Wang et al., 2024; Pathan et al., 2020). These findings are consistent with those reported by Pál & Zsombik (2024), who demonstrated that early incorporation of common vetch green manure increased maize yield by up to 46% under drought conditions in southern Hungary, even outperforming mineral nitrogen fertilization. This emphasizes the potential of vetch-based green manure as a reliable nitrogen source under water-limited Mediterranean environments.

Narbon vetch offers advantages such as high biomass production and drought tolerance (Haffani et al., 2013). However, when incorporated at later phenological stages, increased lignification slows decomposition and delays nitrogen release (Li et al., 2015), which can adversely affect forage quality. Nevertheless, its positive contribution to soil organic matter accumulation renders it valuable for long-term soil health (Pastor-Cavada et al., 2009; Perrone et al., 2020). Enhanced organic matter content improves aggregate stability, soil aeration, water-holding capacity, and root development, while acting as a nutrient reservoir that supports plant growth (Joshna & Bokado, 2024; Shao et al., 2024). Additionally, soils enriched in organic matter promote microbial activity, leading to more efficient nutrient uptake by plants (Abdallah et al., 2024).

Some studies on narbon vetch have shown that phosphorus supplementation and early harvest can improve forage quality by increasing dry matter and crude protein content while reducing fiber levels (Türk, Albayrak & Yüksel, 2007). These results indicate that the limitations associated with narbon vetch can be mitigated with appropriate species selection and optimal timing. Furthermore, its inherent drought resistance allows it to thrive even under water-limited conditions, making it a viable option for sustainable production in arid regions (Tian et al., 2024).

Soil analyses revealed that incorporations made at the early flowering stage resulted in elevated total nitrogen levels, whereas applications conducted at full flowering (100% flowering) were more effective in promoting soil organic matter accumulation. When species were compared, common vetch and forage pea proved more efficient in enhancing soil nitrogen content, while narbon vetch stood out for its superior contribution to organic matter enrichment. The nitrogen supplied through green manure supported protein synthesis, thereby improving both forage yield and quality, while simultaneously acting as a nutrient reservoir for subsequent crops (Dong et al., 2021; Wang et al., 2024).

Green manure applications not only affected soil chemistry but also had significant implications for plant physiology. SPAD values, which are directly linked to leaf chlorophyll content, serve as reliable indicators of photosynthetic efficiency. The incorporation of legume-based green manures enhanced soil nitrogen content, thereby stimulating chlorophyll synthesis and improving plant vigor (Islam et al., 2018). Such strategies resulted in significant increases in SPAD values in subsequent crops, indicating enhanced light interception capacity (Mooleki et al., 2016).

Likewise, the effect of green manure on leaf area index, a robust indicator of plant growth and biomass accumulation, is well documented. Incorporation of species such as common vetch improved soil structure and nitrogen availability, supporting root development and subsequently increasing LAI values (Cho et al., 2015; Kumar et al., 2023; Neely et al., 2024).

Digital indices such as the Normalized Difference Vegetation Index (NDVI) have also proven to be effective tools in monitoring field-level impacts of green manure treatments. The enhanced nitrogen profile and increased canopy density resulting from green manure led to higher NDVI values, reflecting greater green cover (Lee et al., 2015). The nitrogen released through legume biomass decomposition provides a strong signal in early-season monitoring by contributing to denser, greener canopies (Islam et al., 2018; Mukiibi, Franke & Steyn, 2023; Qi et al., 2024).

Various studies have confirmed that green manure applications enhance not only crop yield but also forage quality. Nutrients released from decomposing legume biomass positively influenced crude protein content and digestibility, which are crucial for livestock nutrition and the nutritive value of the forage (Żołnowski et al., 2022; Baath et al., 2018). These physiological improvements demonstrate that green manure applications contribute significantly not only to agricultural productivity but also to the sustainability of forage production systems.

The optimal incorporation period for green manures is typically between flowering and early pod formation (Tigka et al., 2016; Sajjad, 2019). During this window, nitrogen content is high, the C:N ratio is ideal for decomposition, and lignification has not yet occurred. Applications made during this phase enhance nitrogen release, allowing the following crop to benefit maximally (Lynge et al., 2022; Song et al., 2024). Evaluated in terms of physiological indicators, applications at this timing have been shown to improve SPAD, LAI, and gas exchange parameters, which are directly linked to plant growth and biomass production (Akbar et al., 2024).

Incorporating species such as common vetch and forage pea at early developmental stages offers a climate-smart, environmentally friendly alternative to synthetic fertilizers, with clear benefits for both yield and forage quality. The increase in organic matter and microbial diversity also supports improved drought tolerance, water use efficiency, and overall soil biological health (Mokarram-Kashtiban et al., 2019). However, the integration of these strategies into agricultural systems depends on logistical factors such as seed availability, biomass management, crop rotation planning, and incorporation timing. The high seed cost of certain legume species may present economic challenges, making cost-benefit analyses essential for informed decision-making at the producer level. Moreover, since this study was conducted at a single location, its findings may have limited generalizability across varying environmental conditions. Therefore, further multi-site and multi-year trials, complemented by economic evaluations, are crucial for the broader adoption and successful implementation of these sustainable practices.

## Conclusions

This study highlights that the incorporation timing and legume species used as green manure significantly affect the yield, forage quality, and physiological responses of sorghum × sudangrass. Among the treatments, common vetch incorporated at the pre-flowering and 10% flowering stage stood out with its superior performance in fresh and dry biomass, plant height, leaf development, and crude protein content, closely approaching the effects of mineral fertilization. Meanwhile, narbon vetch at early to full flowering produced the highest biomass and was particularly effective in improving soil organic matter and total nitrogen content, albeit with comparatively higher fiber levels.

These findings suggest that for farmers targeting high forage quality and yield, early incorporation of legumes is a promising strategy. Conversely, those focusing on long-term soil fertility may benefit from later incorporation of narbon vetch. Overall, this research supports the use of species- and stage-specific green manure strategies as a sustainable approach to enhance both crop productivity and soil health under Mediterranean conditions.

## Supplemental Information

10.7717/peerj.20137/supp-1 Supplemental Information 1 Raw Dataset. Data for all examined variables and soil data.

## Additional Information and Declarations

### Data Availability

The following information was supplied regarding data availability:

The raw data is available in the Supplemental File.
